# Competitive displacement and acaricide resistance of two *Rhipicephalus* (*Boophilus*) species collected on commercial farms in South Africa

**DOI:** 10.1007/s10493-023-00871-7

**Published:** 2023-12-19

**Authors:** Ellie M. S. P. van Dalen, Candice Jansen van Rensburg

**Affiliations:** Department of Zoology & Entomology, University of the Free State, PO Box 339, Bloemfontein, Free State South Africa

**Keywords:** Amitraz, Cypermethrin, Chlorfenvinphos, *Rhipicephalus decoloratus*, *Rhipicephalus microplus*, Displacement, Resistance

## Abstract

*Rhipicephalus* (*Boophilus*) *microplus*, an invasive species to Africa, and the endemic* R.* (*B.*) *decoloratus* are of high economic importance in the cattle industry. Invasion of the alien species in South Africa has mostly been reported for traditional communal grazing areas where it seemed to be rapid and, in some cases, even replaced the native species. The alien species is also assumed to already be resistant to acaricides upon invasion. The presence of *R.* (*B.*) *microplus* on commercial farms was therefore investigated and resistance screening of both species to field concentrations of cypermethrin, amitraz, and chlorfenvinphos was determined by means of the larval immersion test. Results showed that only 3.7% (of 383) tick collections submitted were *R.* (*B.*) *microplus* populations. A further 1.6% (of 383) showed co-existence of the two species. Comparing the level of resistance to the acaricides between the two species indicated a mean phenotypic resistance of 66.2 and 26.5% of *R.* (*B.*) *decoloratus* populations to cypermethrin and amitraz, respectively. This was significantly lower for *R.* (*B.*) *microplus*, with 23.0 and 4.1% of its populations resistant to cypermethrin and amitraz, respectively. Closed commercial farming areas seemed to have a preventative advantage for the invasion of *R.* (*B.*) *microplus* and displacement of *R.* (*B.*) *decoloratus* by *R.* (*B.*) *microplus*. Regular monitoring of these two species may be of high importance to prevent unnecessary financial losses due to insufficient control and increased awareness of the threat of Asiatic babesiosis vectored by *R.* (*B.*) *microplus.*

## Introduction

*Rhipicephalus* (*Boophilus*) *microplus* (Canestrini) (Acari: Ixodidae) is one of the most economically important tick species worldwide and is also known as the Asiatic or pantropical tick (Horak et al. 2018) or southern cattle tick (Robbertse et al. 2016). In South Africa, Howard initially reported *R.* (*B.*) *microplus* near King Williamstown in the Eastern Cape Province in 1908. Isolated pockets of this species were later recorded in the southern parts of the Western Cape Province (Howell et al. 1978). More recent reports indicated its presence and invasion in some areas, mostly communal grazing areas, found in the Limpopo (Tønnesen et al. 2004), Eastern Cape (Ntondini et al. 2008; Horak et al. 2009; Nyangiwe et al. 2013), Northwest (Spickett et al. 2011) and Mpumalanga provinces (Robbertse et al. 2016). Communal grazing areas are common to African traditions where cattle, belonging to different owners, graze on unfenced communal grounds that do not represent a closed farming system. The cattle are normally not fed any additional concentrates, and in some cases, pastures tend to be overgrazed (Tønnesen et al. 2004). This is in contrast with commercial farms, where producers regulate the cattle herd and cattle of one owner are confined to one fenced-in farm (Bryson et al. 2002).

The closely related African blue tick, *Rhipicephalus* (*Boophilus*) *decoloratus* (Koch), is considered indigenous to Africa and South Africa. Although possible displacement of *R.* (*B.*) *decoloratus* by *R.* (*B.*) *microplus* seems to be evident in some communal areas (Tønnesen et al. 2004; Ntondini et al. 2008; Nyangiwe et al. 2013; Robbertse et al. 2016), *R.* (*B.*) *decoloratus* is still found to be more abundant on commercial farms in South Africa (Horak 1999; Schroder and Reilly 2013). A National Tick Resistance Survey (NTRS) in South Africa, conducted on commercial farms from 1998 to 2001, only recorded four populations of *R.* (*B.*) *microplus* compared to 180 *R.* (*B.*) *decoloratus* populations (Van Dalen and Jansen van Rensburg 2023a). However, Van Wyk et al. (2016) did report co-infestation of both species at 40% of 108 farms in a study where ticks were collected to investigate molecular pyrethroid resistance. The real threat and extent of invasion of *R.* (*B.*) *microplus* on commercial grazing fields of South African cattle do exist but still needs to be elucidated.

Both tick species are of economic importance due to the direct damage both can cause to their hosts. This includes lowered meat and milk production, host anaemia, and damage to hides and teats inflicted by its blood-feeding nature (Jongejan and Uilenberg 2004). The ability of these ticks to act as disease vectors causing babesiosis in its hosts (De Vos 1979; De Vos and Jorgensen 1992) is, however, the main cause of concern that makes information on the distribution of the two species of great importance. *Rhipicephalus* (*B.*) *decoloratus* is the vector for transmission of the pathogen *Babesia bigemina* to cattle during feeding, but *R.* (*B.*) *microplus* acts as a vector for both *B. bigemina* and *Babesia bovis* (Walker et al. 2003). Babesiosis presents in the host with anemia, fever, haemoglobinuria, and splenomegaly. *Babesia bovis* infection, however, seems to be more severe and fast working, as well as being associated with severe nervous system disorders. Animals infected with this pathogen can die soon after symptoms are visible, mostly before treatment can be administered (Bock et al. 2004).

A further factor influencing the economic impact of these two tick species is the development of resistance to chemical control measures (Rajput et al. 2006). Information on the global resistance development of *R.* (*B.*) *microplus* to most classes of acaricides is well-documented (De la Fuente et al. 2000; Rajput et al. 2006; Li et al. 2007; Rodríguez-Vivas et al. 2011; Abbas et al. 2014). The extent and comparison of the expression of phenotypic resistance of these two species to acaricides in South Africa is outdated and more recent information is needed to plan for future control.

Phenotypic resistance of *R.* (*B.*) *decoloratus* to the acaricides initially used for tick control in South Africa has been reported for arsenic (1937), DDT (1954), cyclodiene and toxaphene (1948) organophosphorus and carbamate (1966), pyrethroids (1987), and formamidines (1997) (George et al. 2004). Organophosphate-resistant *R.* (*B.*) *decoloratus* populations (Baker et al.^1978^), as well as populations resistant to pyrethroids, including cypermethrin (Coetzee et al. 1987), were later found on cattle from communal areas located in the Eastern Cape Province. The number of populations tested was, however small and only an indication of the presence and not the extent of tick resistance in the province. A NTRS carried out in South Africa at the turn of the century, showed 35.5% of the *R.* (*B.*) *decoloratus* populations tested to be resistant to cypermethrin, 36.1% to chlorfenvinphos, and 6.6% to amitraz (Van Dalen and Jansen van Rensburg 2023a).

Field data and validation of phenotypic resistance development of *R.* (*B.*) *microplus* in Africa and South Africa are relatively limited. A list with an overview of species displaying acaricide resistance (George et al. 2004) showed reports of resistance of *R.* (*B.*) *microplus* to DDT, cyclodienes, and toxaphene and the organophosphate (OP)—carbamate group in South Africa in 1979. Resistance of *Rhipicephalus* (*Boophilus*) species to formamidines was reported in 1997 and although the resistance of *R.* (*B.*) *decoloratus* to pyrethroids was reported in 1987, no mention of resistance of *R.* (*B.*) *microplus* to this acaricide was noted (George et al. 2004). Ntondini et al. (2008) reported *R.* (*B.*) *microplus* populations resistant to amitraz at three of 45 different dip tanks, resistant to cypermethrin at one of 45 dip tanks, and resistant to chlorfenvinphos at eight of 36 dip tanks tested for resistance during their survey. These dip tanks were all located on communal grounds in the eastern region of the Eastern Cape Province, where herds from different owners were dipped according to a set schedule (Ntondini et al. 2008). A few years later, Lovis et al. (2013) reported that both *R.* (*B.*) *microplus* populations obtained from the eastern parts of the Western Cape Province—one from a commercial and one from a communal farming system—were both susceptible to all the compounds tested. One population obtained from communal grounds in the north-eastern part of Mpumalanga Province was resistant to pyrethroids (Lovis et al. 2013).

Therefore, the present study aimed to provide field data results on the extent of displacement of *R.* (*B.*) *decoloratus* by *R.* (*B.*) *microplus* on commercial farms in South Africa. The phenotypic resistance profiles of *R.* (*B.*) *microplus* and *R.* (*B.*) *decoloratus* was compared and the possible influence of resistance on the invasion of *R.* (*B.*) *microplus* on the farms where they co-existed was investigated. The evolution of phenotypic resistance of *R.* (*B.*) *decoloratus* to different acaricides tested has been described in detail in Van Dalen and Jansen van Rensburg (2023b).

## Materials and methods

### Study area

**Fig. 1:**
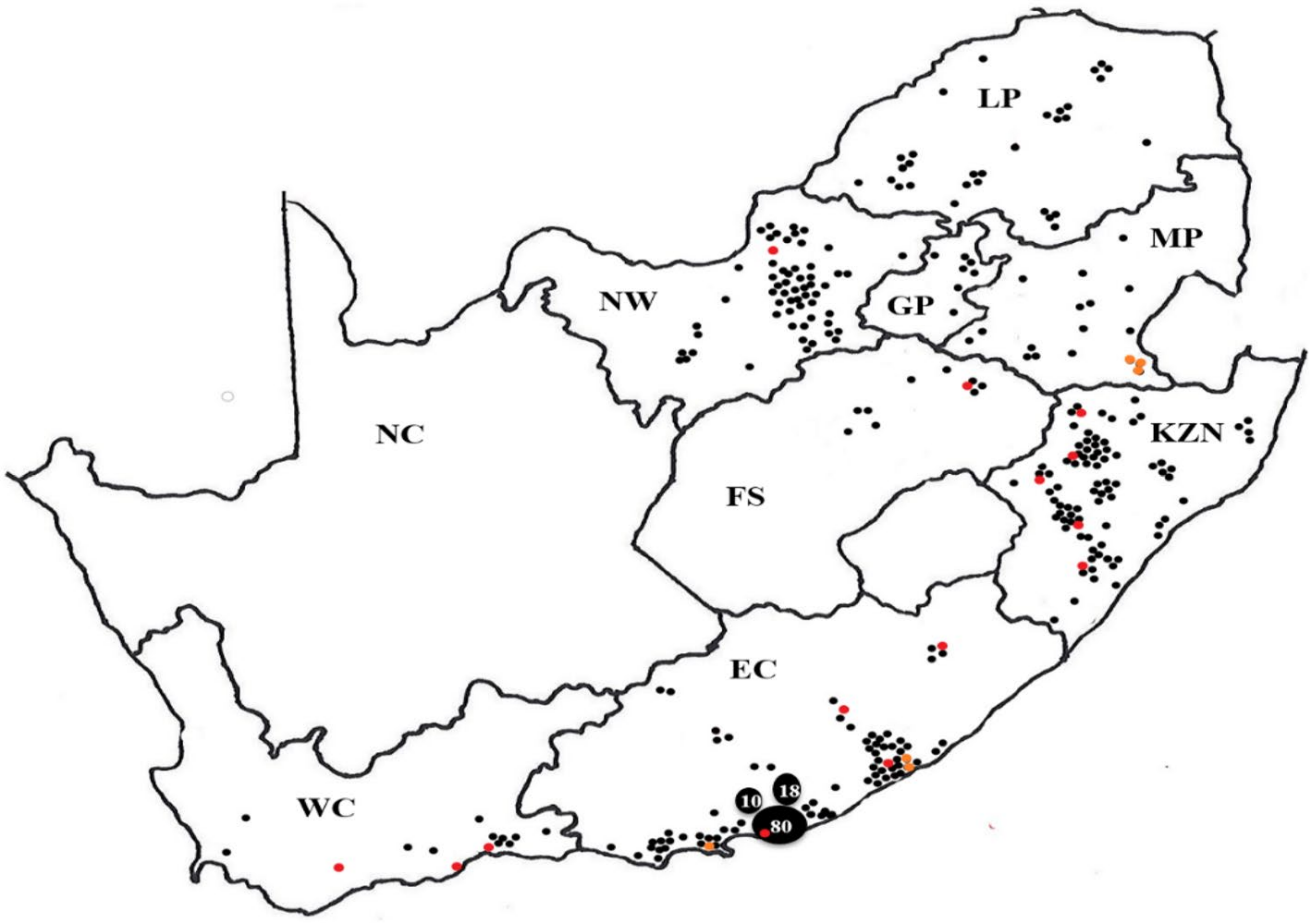
Map of collection points of field populations of *Rhipicephalus* (*Boophilus*) *decoloratus* (black dots), *R.* (*B.*) *microplus* (red dots) and localities where both species were found (orange dots) in the various South African provinces from 2006 to 2017. Provinces: FS, Free State; KZN, KwaZulu-Natal; MP, Mpumalanga; LP, Limpopo; EC, Eastern Cape; NW, Northwest; GP, Gauteng; WC, Western Cape; NC, Northern Cape

Cattle producers and pharmaceutical companies in South Africa randomly submitted *Rhipicephalus* (*Boophilus*) collections from all over South Africa to the Pesticide Resistance Testing Facility (PRTF) situated at the Department of Zoology and Entomology at the University of the Free State in Bloemfontein, South Africa. These populations were only obtained from commercial farms, representing closed farming systems as limited information on this practice is available. Communal farming practices, also found in South Africa, were excluded from this study due to the open cattle grazing practices followed and existing information available in these areas. Populations received from 2006 to 2017 were evaluated as reflected in Fig. 1.

### Experimental procedure

Fully engorged female ticks were collected from the body and dewlap areas of at least 10 cattle on each farm. Individuals from *Rhipicephalus* (*Boophilus*) populations received were identified as either *R.* (*B.*) *decoloratus* or *R.* (*B.*) *microplus* for each population*.* The distinction between the species was made microscopically by using the dentition differences between the two species (Walker et al. 2003). *Rhipicephalus decoloratus* has a dental organization in rows found in 3 × 3 columns on the hypostome, whereas *R.* (*B.*) *microplus* has a 4 × 4 column dental organization. When present in the same collection, the two species were incubated in two separate Erlenmeyer flasks at a temperature ranging between 25 and 28 °C and > 75% relative humidity to allow for oviposition and hatching of the larvae.

The Larval Immersion Test (LIT), developed by Shaw (1966) and most consistently used in South Africa to evaluate tick resistance (Coetzee et al. 1987; Mekonnen et al. 2002; Ntondini et al. 2008), was employed to test for resistance to amitraz, cypermethrin, and chlorfenvinphos. The full description of the methodology used, as adapted by the PRTF, can be found in Van Dalen and Jansen van Rensburg (2023b). Commercially available cypermethrin found in Curatik (15% m/v, 2006–2008) and Pro-dip (20% m/v, 2008–2017); amitraz found in Triatix 125 (12.5% m/v, 2006–2017) and chlorfenvinphos found in Disnis NF dip (9% m/v, 2006–2008) and Coopers Supadip (30% m/v, 2009–2017) were used for acaricide exposure of the larvae of the different populations. Acaricide concentrations were assumed to be correct as indicated on the packaging.

Field concentrations recommended for each acaricide were prepared from an initial 1% m/v, commercial acaricide stock solution to obtain exposure concentrations of 0.025% m/v for amidines, 0.015% m/v for pyrethroids, and 0.05% m/v for organophosphates. These field concentrations were considered to be sufficient to kill > 80% of the individuals in the population as it was assumed to be at least equal to the discriminating concentration to which efficacy needs to be determined. This supposition originated from the conjecture that the correct application of any acaricide remedy at field concentration, registered at the pharmaceutical regulatory body in South Africa, should be effective with at least an 80–100% kill rate. Tick populations exposed to field concentrations of these acaricides should, therefore, fall into this category to be considered susceptible. Populations were further categorized as resistant when the mortality was between 0 and 49%, and emerging resistant between 50 and 79%. Fifty % efficacy was considered to be the tipping point between emerging resistance and resistance as 50% of the individuals in the population were still considered to be susceptible. Producers were, however, warned that urgent action should be taken when the population falls into the emerging resistance category. This evaluation system was devised to accommodate a more economic testing method where a reference strain and a resistance ratio was not taken into consideration to save time, labor and money.

Seventy-two h after exposure of the larvae to a specific acaricide, the live vs. dead larvae were counted to calculate the efficacy of the acaricide for control of the specific population. Larvae from each population were exposed to all three acaricides to also determine multi-resistance.

### Data analysis

Corrections due to incidental mortalities were calculated by using Abbott’s formula where the mean of the duplicate tests was compared to the mean of the control samples to account for control mortalities (Abbott 1987). Only assays with control values of < 10% mortality were included in the results. A population was considered resistant when the mortality was between 0 and 49%, emerging resistant between 50 and 79%, and susceptible between 80 and 100%. The Microsoft Excel 2010 data analysis package was used for statistical analyses and the difference in species frequency was tested with a two-sample t-test assuming unequal variance (α = 0.05).

### Safety measurements

All ticks and larvae were contained and tested in a Section 20 accredited laboratory that assured the safe collection, transport, and handling of ticks to control animal disease. Ethical clearance (No 25/2011A) was obtained from the Interfaculty Animal Ethics Committee of the University of the Free State on a meeting held on 23 February 2012.

## Results

### Distribution of the *Rhipicephalus* (*Boophilus*) species collected

*Rhipicephalus* (*Boophilus*) populations were submitted from commercial farms located along the south-eastern to eastern coastal regions and then inland towards the north and the north-west of South Africa (Fig. 1). *Rhipicephalus* (*B.*) *decoloratus* populations were received from all provinces in South Africa except the Northern Cape Province (Fig. 1, Table 1). *Rhipicephalus* (*B.*) *microplus* collections were submitted from all provinces except Northern Cape, Gauteng, and Limpopo provinces. A significantly higher frequency of *R.* (*B.*) *decoloratus* than *R.* (*B.*) *microplus* populations was submitted to the facility over a 12-year period as tested with a two-sample t-test assuming unequal variance (t = 7.08, d.f. = 10, P < 0.05). The total number of collections received over 12 years (389), contained *R.* (*B.*) *decoloratus* populations on 369 (94.9%), *R.* (*B.*) *microplus* on 14 (3.7%), and both species co-existed on six farms (1.6%) (Table 1).

**Table 1:** The number (% in parentheses) of populations found to be susceptible (S), emerging resistant (ER), and resistant (R) to amitraz, cypermethrin and chlorfenvinphos for *Rhipicephalus* (*Boophilus*) *decoloratus* and *R.* (*B.*) *microplus* in South Africa

Species	No. populations received	Cypermethrin			Amitraz			Chlorfenvinphos		
		S	ER	R	S	ER	R	S	ER	R
*R.* (*B.*) *decoloratus*	369	36 (9.8)	59 (16.0)	274 (74.3)	206 (55.8)	78 (21.1)	85 (23.0)	287 (77.8)	48 (13.0)	34 (9.2)
*R.* (*B.*) *microplus*	20	14 (70)	3 (15)	3 (15)	19 (95)	0 (0)	1 (5)	19 (95)	0 (0)	1 (5)

Seven of the 20 *R.* (*B.*) *microplus* collections were submitted from commercial farms in the Eastern Cape Province located near Cathcart (1), Alexandria (1), Nqanqarhu (previously Maclear) (1), East London (1), Ntabozuko (previously Berlin) (2), and Gqeberha (previously Port Elizabeth) (1). The two collections from farms near Ntabozuko and one from Gqeberha each had co-existing *R.* (*B.*) *decoloratus* populations (Table 2). Five *R.* (*B.*) *microplus* collections from KwaZulu-Natal were submitted from farms close to Bergville, Glencoe, Ixopo, New Castle, and Mooi River (Table 2). Three *R.* (*B.*) *microplus* populations received from the Western Cape Province were submitted from locations near George, Mosselbay, and Swellendam. The *R.* (*B.*) *microplus* population submitted from the Free State Province was found near Vrede and one from the Northwest Province, near Lichtenburg (Table 2). All three collections received from the Mpumalanga Province consisted of *R.* (*B.*) *decoloratus* and *R.* (*B.*) *microplus* and were found on three farms in the Piet Retief area (Table 2).

**Table 2:** Resistance profiles of *Rhipicephalus* (*Boophilus*) *microplus* and *R.* (*B.*) *decoloratus* to cypermethrin (CM), amitraz (AM) and chlorfenvinphos (CFVP)

Province (no. received)	District	*R. decoloratus*			*R. microplus*		
		CM	AM	CFVP	CM	AM	CFVP
Mpumalanga (17)	Piet Retief 1	R	R	S	S	S	S
	Piet Retief 2	ER	S	S	S	S	S
	Piet Retief 3	S	S	S	S	S	S
Eastern Cape (159)	Gqeberha	R	R	S	ER	S	S
	Ntabozuko 1	R	ER	S	S	S	S
	Ntabozuko 2	S	S	S	S	S	S
	Cathcart	–	–	–	S	S	S
	Alexandria	–	–	–	R	R	R
	Nqanqarhu	–	–	–	S	S	S
	East London	–	–	–	S	S	S
Free State (10)	Vrede	–	–	–	S	S	S
Gauteng (7)	No *R.* (*B.*) *microplus*	–	–	–	–	–	–
KwaZulu-Natal (85)	Bergville	–	–	–	S	S	S
	Glencoe	–	–	–	S	S	S
	Ixopo	–	–	–	ER	S	S
	Mooi River	–	–	–	ER	S	S
	New Castle	–	–	–	S	S	S
Limpopo (30)	No *R.* (*B.*) *microplus*	–	–	–	–	–	–
Northwest (59)	Lichtenburg	–	–	–	R	S	S
Western Cape (16)	George	–	–	–	S	S	S
	Mosselbay	–	–	–	S	S	S
	Swellendam	–	–	–	R	S	S

Numbers in parentheses indicate the total number of *R.* (*B.*) *decoloratus* populations collected without the presence of *R.* (*B.*) *microplus* for a specific province. Resistance profile: R, resistant (% efficacy < 50%); ER, emerging resistance (% efficacy 50–79%); S, susceptible (% efficacy > 80%)

### Acaricide resistance profile

Of the 20 *R.* (*B.*) *microplus* populations tested, 70% were susceptible to all three acaricides compared to the 9.8% of 369 *R.* (*B.*) *decoloratus* populations tested (Table 1). Three of the 20 *R.* (*B.*) *microplus* populations were tested as resistant, and a further three as emerging resistant to cypermethrin (Table 2). One of these populations, received from a farm near Alexandria in the Eastern Cape Province, was resistant to all three acaricides tested. This was the only population where resistance of *R.* (*B.*) *microplus* to amitraz and chlorfenvinphos was found (Table 2).

**Fig. 2:**
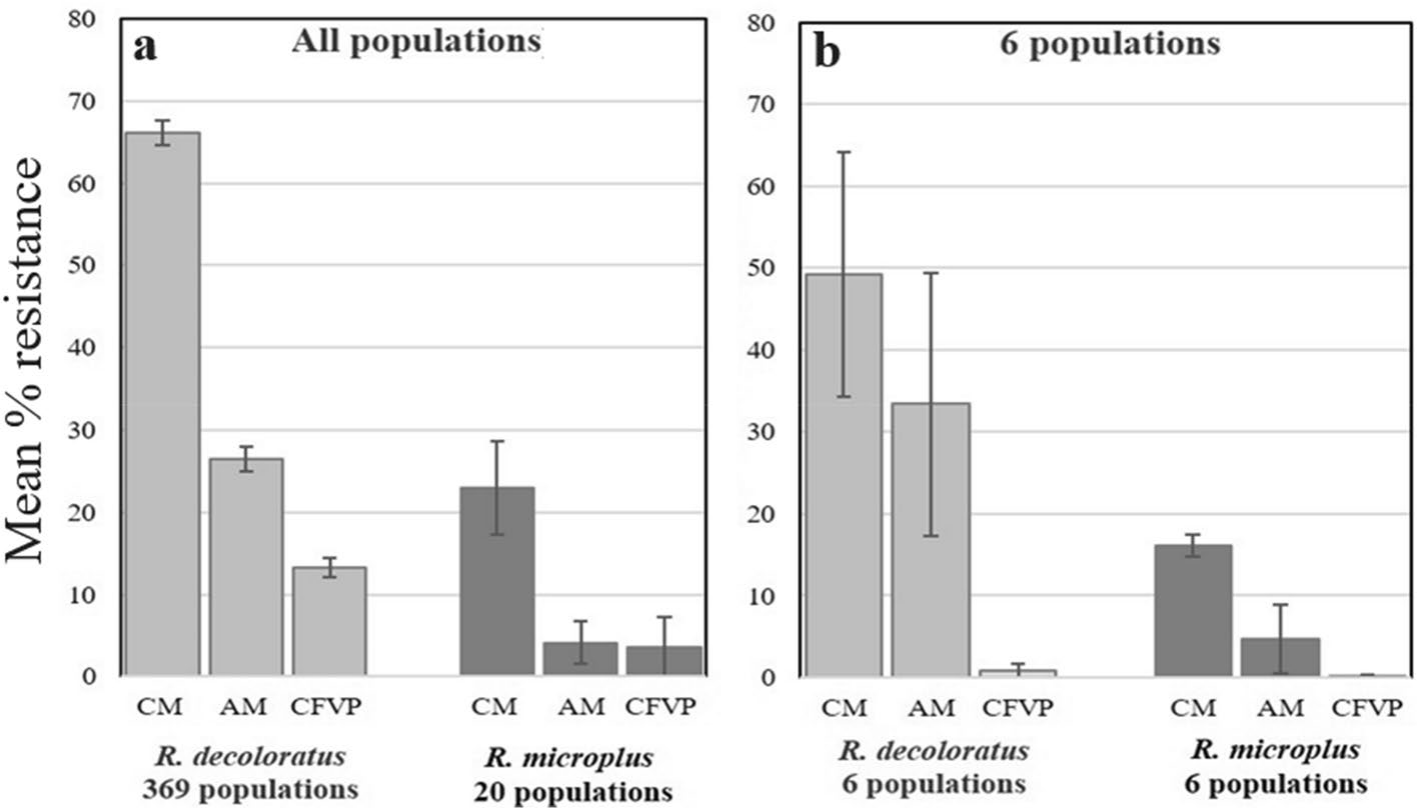
Comparison of the mean (± SE) resistance (%) calculated for (**a**) all the *Rhipicephalus* (*Boophilus*) *decoloratus* vs. *R.* (*B.*) *microplus* populations received from 2006 to 2017 in South Africa, and (**b**) the six populations where both *R.* (*B.*) *decoloratus* and *R.* (*B.*) *microplus* were present in the same blue tick collection obtained from a specific farm in South Africa

A comparison of the level of resistant populations to the acaricides between the two species tested showed that the mean percentage phenotypic resistance of *R.* (*B.*) *decoloratus* to cypermethrin (66.2%) and amitraz (26.5%) was significantly higher than for *R.* (*B.*) *microplus* (cypermethrin 23.0%, amitraz 4.1% and chlorfenvinphos 3.6%) (Fig. 2). This was found when the percentage resistance of all the *R.* (*B.*) *decoloratus* populations tested (369) was compared to the 20 *R.* (*B.*) *microplus* populations received (Fig. 2a), as well as when the two species from the six co-existing localities were compared (Fig. 2b). Mean percentage resistance to chlorfenvinphos was significantly higher for *R.* (*B.*) *decoloratus* (13.2%) when the 369 populations of this species were compared to the 20 *R.* (*B.*) *microplus* (3.6%) populations received. However, when the six co-existing populations were compared, no significant difference was found (Fig. 2b), and both species had a mean percentage resistance of < 1%.

## Discussion

Worldwide resistance of *R.* (*B.*) *microplus* to the major chemical classes of acaricides currently in use has been reported (De la Fuente et al. 2000; Li et al. 2007; Rajput et al. 2006; Rodríguez-Vivas et al. 2011). Historically *R.* (*B.*) *microplus* parasitized bovid hosts in India and Indonesia (Barré and Uilenberg 2010; Labruna et al. 2009) and was introduced into South Africa, with imported cattle, via Madagascar after the rinderpest epidemic in 1896 (Theiler 1962).

Many factors can influence the distribution and possible invasion of alien species into an area. The movement of hosts from one area to another, linked with favorable environmental conditions for the invasive species, could possibly be some of the key factors (Tønnesen et al. 2004). One important aspect must however also be considered. Once introduced into an area, tick control practices and selection for resistance to acaricides can play an important role in the rate of invasion of *R.* (*B.*) *microplus* on commercial farms. Although phenotypic resistance was found for *R.* (*B.*) *microplus* in a communal grazing area, Mnisi, located in the Mpumalanga Province (Malan 2015), acaricide resistance of this species had not been extensively tested for commercial farms in South Africa. *Rhipicephalus* (*B.*) *decoloratus*, however, showed a high prevalence of resistance to pyrethroid-based acaricides as well as amidines on commercial farms in South Africa (Van Dalen and Jansen van Rensburg 2023b).

Studies mapping the prevalence of different tick species in South Africa (Bryson et al. 2002; Horak et al. 2009, 2015; Tonetti et al. 2009; Spickett et al. 2011; Nyangiwe et al. 2017), showed that *R.* (*B.*) *microplus* was present in all provinces in localized areas. The rapid spread of *R.* (*B.*) *microplus*, when introduced to new areas, throughout Africa (Madder et al. 2007, 2011; Adakal et al. 2013), has also been reported for communal areas in South Africa by Ntondini et al. (2008), Horak et al. (2009) and Nyangiwe et al. (2013). This led to the questions whether this total invasion was also true for commercial farms in South Africa? And if not, what prevented invasion of this alien species, seen in the light that the first reports of *R.* (*B.*) *microplus* was already made in 1908 (Howard 1908) and the rapid spread of this species, once introduced, was seen in other African countries (Madder et al. 2007, 2011; Adakal et al. 2013). Surprisingly *R.* (*B.*) *microplu*s populations were only received from 3.7% of the commercial farms that submitted blue tick populations during the current study.

Although represented by low percentages, the prevalence, and localities where *R.* (*B.*) *microplus* populations were found, agreed with localities of previous reports of its presence over the years. In the Western Cape province isolated pockets of *R.* (*B.*) *microplus* were previously reported by Howell et al. (1978) and more recently by Nyangiwe et al. (2017) in corresponding areas along the southern coast where *R.* (*B.*) *microplus* in the current study were collected on three commercial farms. In the Free State Province, only one dominant *R.* (*B.*) *microplus* population was found on a farm near Vrede situated in the northeast of the province where Horak et al. (2015) and Nyangiwe et al. (2017) also previously confirmed the presence of *R.* (*B.*) *microplus*. Baker et al. (1967) reported *R.* (*B.*) *decoloratus* to be one of four species that were most prevalent in KwaZulu-Natal without mentioning the presence of *R.* (*B.*) *microplus* in this survey. In 2014 Oberholster reported a 60% presence of *R.* (*B.*) *decoloratus* on farms in KwaZulu-Natal, with a discontinuous distribution of *R.* (*B.*) *microplus* in temperate parts of the province corresponding to the areas where five dominant *R.* (*B.*) *microplus* populations were collected during the current study. Both Bryson et al. (2002) and Spickett et al. (2011) reported limited distribution of *R.* (*B.*) *microplus* in the north-western areas of the Northwest Province. Oberholster (2014) found *R.* (*B.*) *decoloratus* to be more prevalent than *R.* (*B.*) *microplus* on commercial farms, in this province matching the results of the current study. The absence of *R.* (*B.*) *microplus* was ascribed to the more arid climatic conditions of this province (Oberholster 2014).

In Mpumalanga province Malan (2015) reported only the presence of *R.* (*B.*) *microplus* on cattle from a communal dipping system in the Mnisi community, bordering the Kruger National Park in the northeastern part of the province. The three collections of co-existing *Rhipicephalus* (*Boophilus*) species collected in the current study were, however, found on commercial farms in the southeastern part of the province which were quite a distance from Mnisi. This presence of *R.* (*B.*) *microplus* must have represented another introduction of this species than possible closeby introduction as was found in the previous provinces.

In the Limpopo province, Tønnesen et al. (2004) reported the presence of *R.* (*B.*) *microplus* at 93.4% vs. the 6.6% of *R.* (*B.*) *decoloratus* at dip tanks during a survey done from 1999 to 2001. They also found *R.* (*B.*) *microplus* to co-exist with *R.* (*B.*) *decoloratus* on two of the five commercial farms investigated in this province. The current study did, however, not yield any *R.* (*B.*) *microplus* populations on any of the 30 commercial farms investigated in both this province and the seven for Gauteng province.

In an attempt to link invasion with resistance, the results obtained from the Eastern Cape province may be of importance. Initially, *R.* (*B.*) *microplus* was only found in communal areas east of East London in the eastern part of this province (Ntondini et al. 2008; Horak et al. 2009). In the current study, three of the four *R.* (*B.*) *microplus* populations received, as part of 159 commercial farms investigated, were from the eastern part of this province. Co-existence with *R.* (*B.*) *decoloratus* occurred on a further two farms near Ntabozuko, situated in the more central part closer to East London. These findings can be expected due to possible introduction of *R.* (*B.*) *microplus* on cattle from infested areas close to these farms. If the focus is moved more to the western part of this province, Horak et al. (1999) found only *R.* (*B.*) *decoloratus* on a commercial farm close to Alexandria in a survey carried out from 1982 to 1983. Years later, Nyangiwe et al. (2017) reported the presence of *R.* (*B.*) *microplus* throughout the coastal regions of the western part of the Eastern Cape Province, but Yawa et al. (2019) on the other hand were unable to confirm its presence in the western central regions of this province. In the current study *R.* (*B.*) *microplus* was only present on a farm near Alexandria, indicating a possible introduction, invasion, and eventual displacement of *R.* (*B.*) *decoloratus* on this farm. A high occurrence of multi resistance of *R.* (*B.*) *decoloratus* populations was observed on farms in the Alexandria district over a period of 12 years (Van Dalen and Jansen van Rensburg 2023b).

From this information the steps of displacement can perhaps be explained as follows. The introduction of a susceptible strain of *R.* (*B.*) *microplus* may initially be successfully controlled by the acaricide in use to mask the presence of this tick species. A gradual resistance development of the introduced *R.* (*B.*) *microplus* population may then increasingly outcompete *R.* (*B.*) *decoloratus* individuals on the specific farm, even if they were also resistant, due to the higher level of survival of the chemical onslaught. This assumption is further strengthened by the resistance profile of *R.* (*B.*) *decoloratus* vs. *R.* (*B.*) *microplus* coexisting on another farm in the Gqeberha area close to Alexandria. The acaricide resistance profile of *R.* (*B.*) *decoloratus* indicated multi-resistance to cypermethrin and amitraz, whereas the coexisting *R.* (*B.*) *microplus* indicated resistance to only cypermethrin. This could substantiate the possible first steps of *R.* (*B.*) *microplus* invasion to displacement. The one *R.* (*B.*) *microplus* population, with resistance to all three acaricides tested, and without the presence of a coexisting *R.* (*B.*) *decoloratus* found, could represent the ultimate displacement occurrence where the resistance of both species to these acaricides caused *R.* (*B.*) *microplus* to be the winner in this war. This postulate needs to be further investigated to confirm or deny this possibility.

The ability of *R.* (*B.*) *microplus* to outcompete *R.* (*B.*) *decoloratus* when on equal footing—e.g., both susceptible or both resistant—is grounded in the life cycle advantages of *R.* (*B.*) *microplus* compared to *R.* (*B.*) *decoloratus.* These advantages include a slightly shorter life cycle (Londt and Arthur 1975), the production of a higher number of eggs, and the production of sterile eggs when cross mating between the two species takes place (Spickett and Malan 1978). Cross mating occurs due to the sex ratio favoring males (2:1) (Horak et al. 1992, 2003). Although conspecific mating is preferred (Norval and Sutherst 1986), sexually mature *R.* (*B.*) *microplus* males do mate with both *R.* (*B.*) *microplus* and *R.* (*B.*) *decoloratus* females in mixed populations before *R.* (*B.*) *decoloratus* males can do so, causing *R.* (*B.*) *decoloratus* females to produce sterile eggs (Horak et al. 2009). Nyangiwe et al. (2013) found cross mating of *R.* (*B.*) *microplus* males with *R.* (*B.*) *decoloratus* females but none between *R.* (*B.*) *decoloratus* males and *R.* (*B.*) *microplus* females. They furthermore noticed *R.* (*B.*) *microplus* males clasping engorged *R.* (*B.*) *decoloratus* nymphs while attached next to them and upon dissection of these nymphs discovered that nine out of 10 of these occurrences were destined to molt into females (Nyangiwe et al. 2013).

Review of *Rhipicephalus* (*Boophilus*) species populations received over 12 years in the current study indicated that the level of invasion of *R.* (*B.*) *microplus* and displacement of *R.* (*B.*) *decoloratus* by *R.* (*B.*) *microplus* on commercial farms were still low in comparison to the prevalence of *R.* (*B.*) *decoloratus* populations. These results indicated that commercial farming practices might, to a great extent help to conserve the dominant prevalence of *R.* (*B.*) *decoloratus* on commercial farms. Closed farming practices followed by commercial producers, compared to the greater movement of cattle in communal areas, seem to help limit the spread of *R.* (*B.*) *microplus* to commercial farms even when located close to a communal area. It further suggests that a susceptible strain of *R.* (*B.*) *microplus* may sometimes be introduced onto most commercial farms but is initially successfully controlled by the acaricide in use. This displacement of *R.* (*B.*) *decoloratus* will, however, also occur if the *R.* (*B.*) *microplus* individuals introduced are already resistant to the acaricide in use on the specific farm and might again be controlled if another acaricide is used, for which resistance was not selected. Collections made on communal grounds and tested for susceptibility to cypermethrin and amitraz in the Mnisi community located in the Mpumalanga Province showed the presence of only *R.* (*B.*) *microplus* with a high prevalence of resistance to these acaricides (Malan 2015) and may also confirm that the resistance profile of the invasive species in the end played the dominant role.

When both species are susceptible to acaricide control, *R.* (*B.*) *microplus* populations can outcompete *R.* (*B.*) *decoloratus* (Arthur and Londt 1973; Londt & Arthur 1975; Spickett and Malan 1978) while gradually developing resistance to the acaricide in use. This will, however, be a slow process, as initial effective acaricide control will keep the numbers of both species low. In this case, the race between the two species to obtain resistance to the acaricide in use will more likely be the determining factor. The higher tendency in South Africa to make use of pyrethroid-based acaricides in the past (Van Dalen and Jansen van Rensburg 2023a), can explain the higher occurrence of resistance of *R.* (*B.*) *microplus* to pyrethroids, compared to amitraz and chlorfenvinphos resistance on commercial farms investigated. Unfortunately, the treatment history of these farms was not available to come to more specific conclusions.

The problem with resistance is that it could have caused producers to indiscriminately use all available acaricides in short succession, giving *R.* (*B.*) *microplus* populations an advantage over *R.* (*B.*) *decoloratus* for resistance development once introduced on a specific farm.

The close morphological similarities between the two tick species sometimes causes producers to only become aware of the presence of *R.* (*B.*) *microplus* during an outbreak of babesiosis caused by *B. bovis* (Tønnesen et al. 2004). This disease has a more severe clinical result than babesiosis caused by *B. bigemina.* The economic consequences of the invasion of *R.* (*B.*) *microplus*, and tick resistance development to chemical control, calls for early detection and monitoring of both the presence and resistance of *R.* (*B.*) *microplus* on commercial farms.

Regular monitoring for tick resistance development on a commercial farm, combined with the identification of the *Rhipicephalus* (*Boophilus*) species found, can be useful tools to plan control strategies. This in turn will prevent increased economic losses due to resistance development to chemical control and Asiatic babesiosis caused by the invasive *R.* (*B.*) *microplus* species as vector. Molecular identification for the presence of mutations causing tick resistance in a population can be useful (Robbertse et al. 2016), but to characterize acaricide resistance in an area or on a farm, conventional testing such as the LIT (Shaw 1966) is still necessary to determine the extent of the resistance development in the population.

## Conclusion

Although the invasion of *R.* (*B.*) *microplus* on communal farms seems to be rapid and, in some cases, replaces the native species in these areas, this phenomenon does not seem to be true for commercial farms in South Africa, at least not for now. Closed commercial farming systems seem to have a preventative advantage over communal grazing systems for the invasion of *R.* (*B.*) *microplus*. A very low percentage of commercial farms showed the presence of *R.* (*B.*) *microplus* populations. This can be ascribed to low introduction of cattle from outside herds that could introduce *R.* (*B.*) *microplus* onto a commercial farm, as well as the susceptible status of the introduced *R.* (*B.*) *microplus* to acaricide control practices on the specific farm.

Once introduced on a commercial farm, the possibility of displacement of *R.* (*B.*) *decoloratus* does exist. The first step after introduction can be total chemical control of *R.* (*B.*) *microplus* with the acaricide in use, and therefore eradication, followed by the gradual resistance development and once both species become resistant to acaricide in use, *R.* (*B.*) *microplus* can start to outcompete *R.* (*B.*) *decoloratus.* Environmental conditions, acaricide control and resistant management practices may play an important role to determine the rate at which *R.* (*B.*) *microplus* might take over.

## Data Availability

The datasets generated during and/or analysed during the current study are available from the corresponding author on reasonable request.
